# Engineering bionanoreactor in bacteria for efficient hydrogen production

**DOI:** 10.1073/pnas.2404958121

**Published:** 2024-07-10

**Authors:** Weiming Tu, Ian P. Thompson, Wei E. Huang

**Affiliations:** ^a^Department of Engineering Science, University of Oxford, Oxford OX1 3PJ, United Kingdom

**Keywords:** H_2_ production, bionanoreactor, *Gloeobacter* rhodopsin, *Shewanella oneidensis* MR-1, nanomaterials

## Abstract

Hydrogen production through water splitting is a vital strategy for renewable and sustainable clean energy. In this study, we developed an approach integrating nanomaterial engineering and synthetic biology to establish a bionanoreactor system for efficient hydrogen production. The periplasmic space (20 to 30 nm) of an electroactive bacterium, *Shewanella oneidensis* MR-1, was engineered to serve as a bionanoreactor to enhance the interaction between electrons and protons, catalyzed by hydrogenases for hydrogen generation. To optimize electron transfer, we used the microbially reduced graphene oxide (rGO) to coat the electrode, which improved the electron transfer from the electrode to the cells. Native MtrCAB protein complex on *S. oneidensis* and self-assembled iron sulfide (FeS) nanoparticles acted in tandem to facilitate electron transfer from an electrode to the periplasm. To enhance proton transport, *S. oneidensis* MR-1 was engineered to express *Gloeobacter* rhodopsin (GR) and the light-harvesting antenna canthaxanthin. This led to efficient proton pumping when exposed to light, resulting in a 35.6% increase in the rate of hydrogen production. The overexpression of native [FeFe]-hydrogenase further improved the hydrogen production rate by 56.8%. The bionanoreactor engineered in *S. oneidensis* MR-1 achieved a hydrogen yield of 80.4 μmol/mg protein/day with a Faraday efficiency of 80% at a potential of −0.75 V. This periplasmic bionanoreactor combines the strengths of both nanomaterial and biological components, providing an efficient approach for microbial electrosynthesis.

## Significance

Hydrogen production through water splitting has been substantially improved by engineering the nanoscale periplasmic space (20 to 30 nm) of *Shewanella oneidensis* MR-1, transforming it into a highly effective bionanoreactor. This design concentrated protons and electrons, enhancing hydrogen production. The integration of reduced graphene oxide (rGO), MtrCAB complex, and iron sulfide (FeS) nanoparticles established an effective electron transfer chain from the electrode to the periplasm. The introduction of *Gloeobacter* rhodopsin and canthaxanthin boosted proton pumping into the periplasm under light. This system, catalyzed by the overexpression of [FeFe]-hydrogenase and powered by both electricity and light, achieved a Faraday efficiency of 80% for hydrogen production. This periplasmic bionanoreactor marks an advancement in sustainable synthetic biology.

In response to the global energy crisis and environmental challenges, there is a growing demand for clean energy sources (1). Hydrogen is the quintessential clean energy solution, distinguished by its high energy density and environmentally friendly combustion properties (2). Green hydrogen, generated by water splitting driven by renewable energy sources, is regarded as the most promising energy carrier for a hydrogen economy (3). The quest for economical, stable, and efficient catalysts for cathodic H_2_ generation in an electrochemical system has attracted wide interest (4). The cost of hydrogen production is the most important challenge hampering deployment (5). Traditional metal materials, commonly used as the cathode for water electrolysis, often entail substantial cost implications (6). In contrast, biocatalysts, such as microbial cells, offer a cost-effective alternative for catalyzing H_2_ synthesis under mild conditions (7).

The metal-reducing bacterium, *Shewanella oneidensis* MR-1 is a model electroactive platform, which is commonly employed for microbial fuel cells (8, 9) and microbial electrosynthesis (10, 11) because of its bidirectional extracellular electron transfer pathway (12, 13). *S. oneidensis* MR-1 can be used as a biocatalyst in the electrochemical system for cathodic H_2_ production by combining its extracellular electron transfer mechanism and hydrogenase-based metabolism (14). *S. oneidensis* MR-1 has encoded two native hydrogenases, namely, [NiFe]- and [FeFe]-hydrogenases, located in the periplasmic space with the capacity to catalyze the interconversion between protons and hydrogen (15). The electrode, as an external electron donor, delivers electrons across the outer membrane into hydrogenases reducing protons to hydrogen. Despite theoretical feasibility, the current wild-type (WT) *S. oneidensis*–based H_2_ electrosynthesis system exhibits unsatisfactorily low hydrogen yield that largely limits the practical applications (14, 16). Hydrogen production is a straightforward reaction, involving the interactions of protons and electrons, catalyzed by hydrogenases in the periplasm. We hypothesized that the poor microbial electrosynthesis of H_2_ is attributed to inefficient electron transport and suboptimal periplasmic conditions.

Low extracellular electron-transfer rate is a common limiting factor for the electrochemical application of *S. oneidensis* MR-1 (12, 17). Considerable efforts have been devoted to enhancing the extracellular electron-transfer processes (18, 19). Introducing electron-shuttling molecules such as flavins (20) and iron ions (21) has been widely used to boost the electrochemical performance of *S. oneidensis* MR-1. Recent strategies focus on coupling the nanoparticle with *S. oneidensis* MR-1, which have been reported to significantly promote extracellular electron transfer (17, 18, 22). *S. oneidensis* MR-1 is a well-established bionanofactory for synthesizing and assembling nanoparticles due to its reducing and detoxification capacities (16, 23). H_2_ synthesis of *S. oneidensis* MR-1 takes place in the narrow periplasmic space which yields the opportunity to rationally design the periplasm as a nanoreactor by optimizing synergy between nanoparticles and biocomponents (24).

In addition to improving electron transport, a high proton concentration could also facilitate the catalytic reaction from protons to hydrogen. It has been reported that expression of a proton-pumping proteorhodopsin (PR) in *Escherichia coli* with the heterologous hydrogenase increased hydrogen production yield by around 30% under light (25). Microbial rhodopsin is a simple protein which can used as an optogenetic tool to engineer nonnative bacteria with light-harvesting capacity (26, 27). Outward proton pumping rhodopsins such as PR (27) and *Gloeobacter* rhodopsin (GR) (28) have been used to enhance microbial energy metabolism while their applications in microbial electrochemical H_2_ production remain largely unexplored. Furthermore, the increase of hydrogenase concentration in the periplasm is expected to boost the reaction rate (29).

**Fig. 1.**
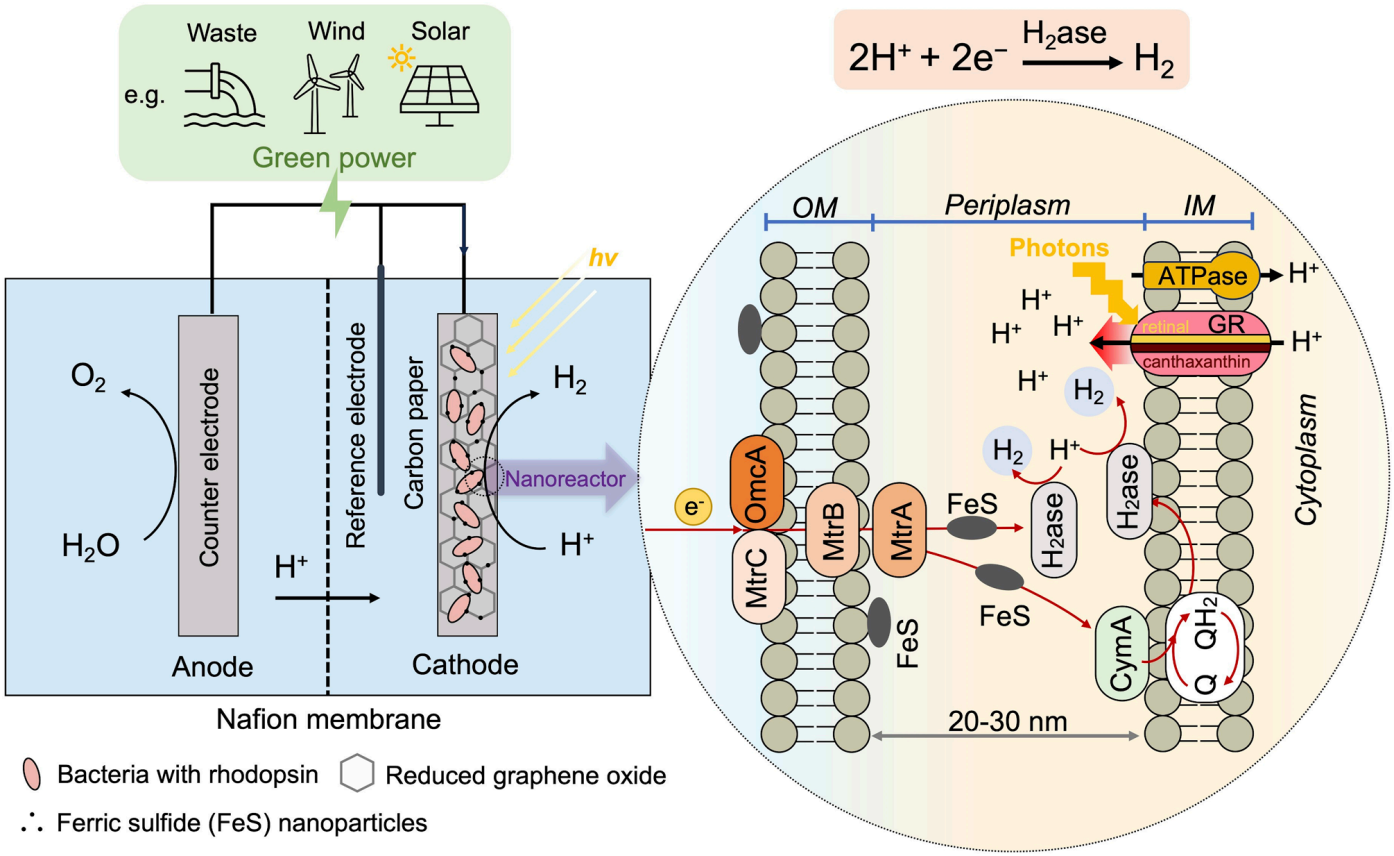
Schematic of the sustainable bioprocess for H_2_ bioproduction. *S. oneidensis* MR-1 uses hydrogenase to catalyze H_2_ synthesis from protons and electrons, powered by light and electricity. Reduced graphene oxide (rGO) and FeS nanoparticles were introduced to enhance the electron transfer. The cells were engineered to express GR and its antenna CAN, which harvest photons to pump protons from cytoplasm to periplasm.

We propose that the efficiency of hydrogen production could be substantially improved by designing a bionanoreactor capable of effectively mediating elevated concentrations of protons, electrons, and hydrogenases. In this study, a periplasmic bionanoreactor based on *S. oneidensis* MR-1 has been designed for H_2_ production (Fig. 1). The use of biologically reduced graphene oxide (rGO) and native MtrCAB protein complex enhanced electron transfer from the electrode into *S. oneidensis* MR-1. *S. oneidensis* MR-1 was also employed to synthesize iron sulfide (FeS) nanoparticles which are self-assembled and located within the periplasm to mediate the electron transfer. FeS nanoparticles play the role of a conductive chain to facilitate the electron transfer from the outer-membrane proteins into hydrogenase at periplasm. In addition, *S. oneidensis* MR-1 was engineered to heterologously express GR and its antenna canthaxanthin (CAN), which was able to harvest light energy to efficiently pump protons from cytoplasm to periplasm. In the periplasm-based bionanoreactor, electrons, protons, hydrogenases, as well as conductive FeS were all concentrated into the nanoscale space of periplasm, facilitating an enhanced H_2_ synthesis. This rationally designed system achieved efficient photoelectrochemical hydrogen production. This proof-of-concept study could offer an insight into engineering bacterial catalysts with nanoparticles and genetic reprogramming for efficient microbial electrosynthesis.

## Results

### Biogenic FeS Nanoparticles Assembled in Periplasm of *S. oneidensis* MR-1 for Enhanced Inward Electron Transfer.

**Fig. 2.**
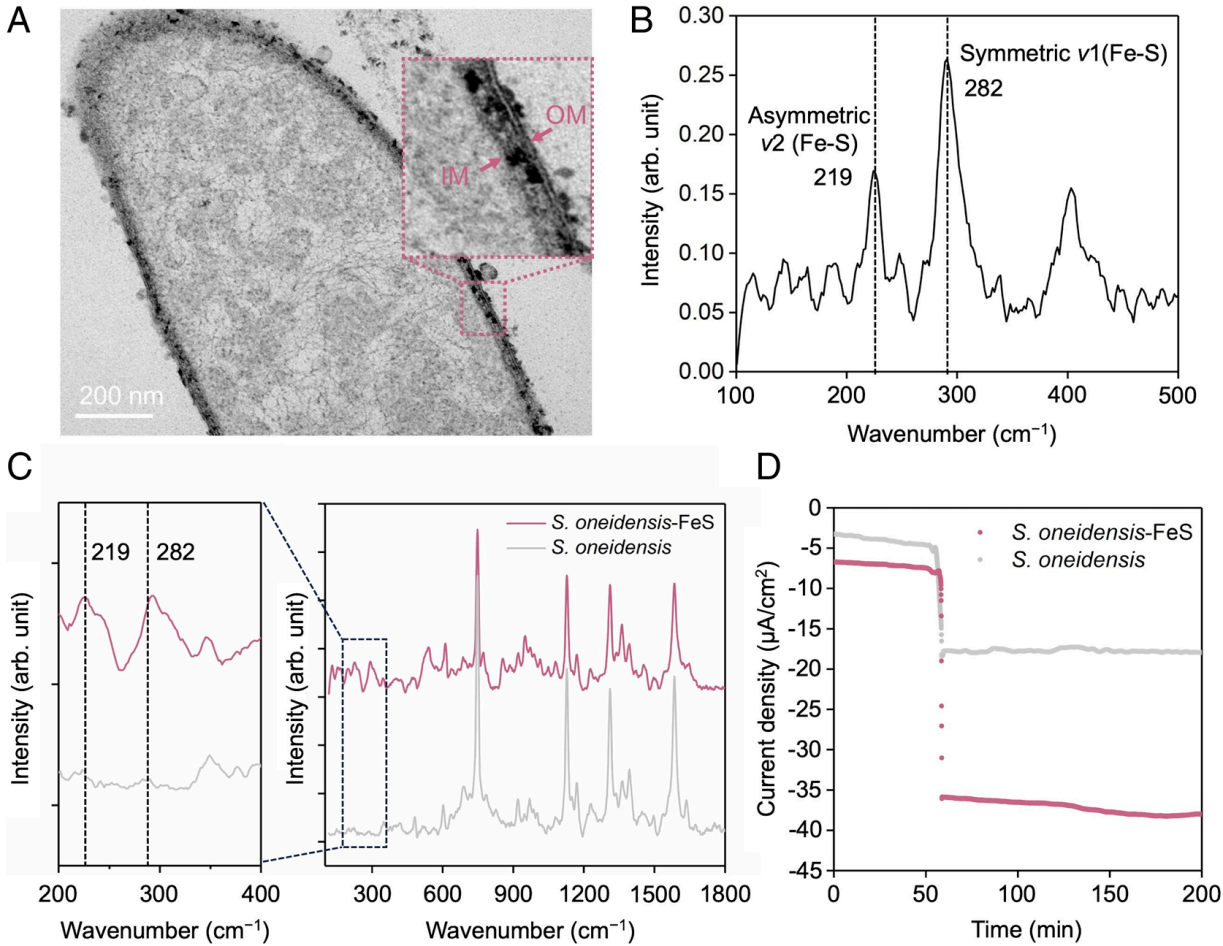
The self-assembly of biogenic FeS nanoparticles in the periplasm of *S. oneidensis* MR-1. (*A*) A TEM image of *S. oneidensis* MR-1 after two-day incubation with thiosulfate and FeSO_4_ to form FeS nanoparticles in the periplasm and on the outer membrane. OM: outer membrane; IM: inner membrane. (*B*) A typical Raman spectrum of FeS nanoparticle synthesized by *S. oneidensis*. (*C*) Single-cell Raman spectra of cells of *S. oneidensis* with and without FeS nanoparticles. (*D*) Fumarate reduction test. The biocathode was poised at −0.5 V (vs. Ag/AgCl), and 25 mM fumarate was added at ~50 min.

*S. oneidensis* cells, as bionanofactories, have been reported to synthesize a variety of multifunctional nanomaterials, such as silver (18) and cadmium nanoparticles (16, 30) by reducing metal salts. In this study, *S. oneidensis* MR-1 was employed to synthesize FeS nanoparticles because of its highly reductive and unique electrical properties along with easily accessible raw materials (31). Transmission electron microscopy (TEM) image of the cell with synthesized FeS shows that the formed nano-sized particles (15.5 ± 5.8 nm) were mostly located in the periplasmic space with a small number of the nanoparticles attached to the outer membrane of cells (Fig. 2*A* and *SI Appendix*, Figs. S1 and S2). Raman microspectroscopy was used to verify the biosynthesized FeS nanoparticles at the single-cell level (Fig. 2*B*). Two characteristic Raman bands at 219 and 282 cm^−1^ were identified in the spectrum of purified FeS biosynthesized by *S. oneidensis* MR-1, and these bands were attributed to the characteristic Fe–S vibrations (32). Single-cell Raman analysis revealed that Raman spectra of the cells assembled with FeS nanoparticles also displayed the bands associated with the Fe–S vibrations (Fig. 2*C*), in a good agreement with the TEM result of *S. oneidensis* MR-1 cells with self-assembled FeS nanoparticles.

From the TEM image, it can be seen that a large number of FeS nanoparticles were located in the periplasm, which could facilitate the periplasmic electron transfer. Electrochemical tests were performed to evaluate the effect of FeS nanoparticles on the inward extracellular electron transfer of *S. oneidensis*. Fumarate was introduced as an electron acceptor into the *S. oneidensis*–based electrochemical system. Under anaerobic cathodic condition at a potential of −0.5 V (vs. Ag/AgCl), 25 mM fumarate was injected into the cathode chamber containing either *S. oneidensis* or *S. oneidensis*-FeS. The addition of fumarate was expected to enhance electron-transfer processes because *S. oneidensis* contains a periplasmic fumarate reductase catalyzing the reduction of fumarate to succinate (12). Monitoring of the current consumption revealed a prompt decrease in the cathodic current for both *S. oneidensis* with and without FeS nanoparticles (Fig. 2*D*). Notably, the *S. oneidensis*-FeS demonstrated over twofold increase in inward current density (−37 μA/cm^2^) compared to *S. oneidensis* without FeS nanoparticles (−17 μA/cm^2^). This result suggests that the self-assembled FeS nanoparticles in the *S. oneidensis*-FeS strain could enhance the inward electron transfer rate.

### Constructing a Biologically Reduced Graphene Oxide with *S. oneidensis* –FeS Nanoparticles for H_2_ Production.

*S. oneidensis* was employed as a biocatalyst to generate H_2_ by hydrogenases (14). Linear scanning voltammetry (LSV) analysis was used to measure hydrogen evolution reaction activity for the carbon paper cathode pregrown with *S. oneidensis* MR-1 at neutral pH. The LSV results show a significant cathodic current with an onset potential of around –0.56 V vs. Standard Hydrogen Electrode (SHE) for the *S. oneidensis* MR-1 biocathode, in contrast to weak reduction currents observed in the control group of the carbon paper-based electrode (*SI Appendix*, Fig. S3*A*). These results suggest that *S. oneidensis* MR-1-based biocathode should catalyze proton reduction with an overpotential of around 0.15 V, compared to the theoretical hydrogen evolution potential of –0.41 V vs. SHE. When the cathodic potential was set at –0.7 V vs. SHE, the biocathode with *S. oneidensis* MR-1 exhibited significant hydrogen production, in contrast to the negligible amount of hydrogen detected in the control without bacteria (*SI Appendix*, Fig. S3*B*).

**Fig. 3.**
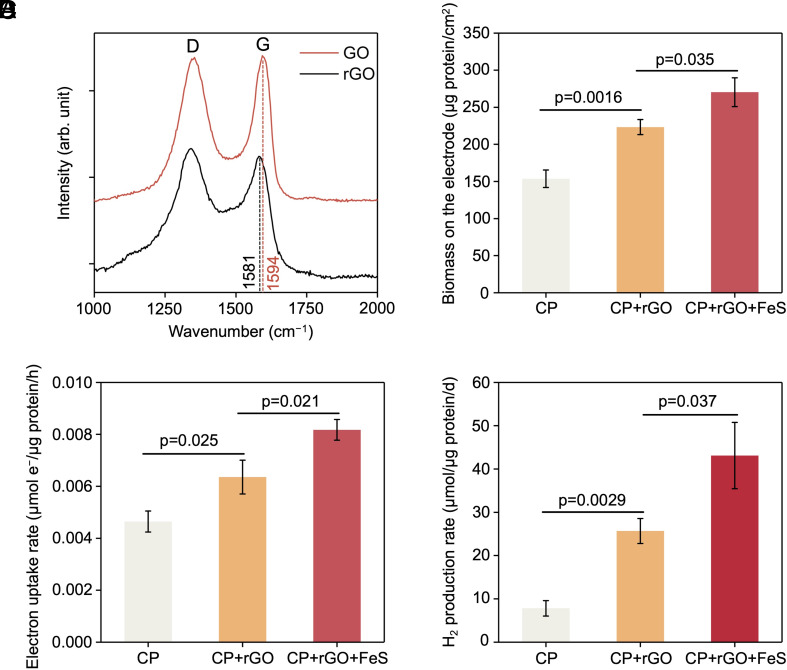
The integration of the biogenic rGO with *S. oneidensis* MR-1 enhanced H_2_ production. (*A*) Raman spectra of GO and rGO resulting from the reduction reaction by *S. oneidensis* MR-1. (*B*) The biomass protein on the electrodes based on carbon paper (CP), CP with rGO, and CP with rGO and self-assembled FeS nanoparticles. (*C*) Average current consumption per cell and (*D*) H_2_ production over 72 h using the bioelectrode based on CP, CP with rGO, and CP with rGO and FeS nanoparticles at the cathodic potential of −0.75 V vs. SHE. Statistics were performed with Student’s *t* test (data are means ± SD, n = 3).

The microbial electrochemical system can be combined with material engineering to enhance H_2_ production. For instance, graphene has been widely applied to enhance the electrochemical properties of the biocathode, due to its high conductivity and large surface area (18, 19). To enhance the electrochemical performance in this study, microbially rGO to coat the electrode was employed. The outer membrane cytochromes of *S. oneidensis* MR-1 have been reported to catalyze graphene oxide (GO) to rGO (33). *S. oneidensis* MR-1 cells were mixed with GO and the mixture was incubated for 24 h at room temperature. Raman spectroscopy was employed to compare the spectra of the *S. oneidensis* MR-1-GO samples before and after the incubation (Fig. 3*A*). The initial GO exhibited two sharp bands. The D band (around 1,350 cm^−1^) was associated with the K-point phonons of A_1 g_ symmetry, and the G band (around 1,580 to 1,600 cm^−1^) was attributed to the zone center phonons of E_2 g_ symmetry (34). An obvious red shift of the G band of the samples, transitioning from 1,594 to 1,581 cm^–1^, was observed after microbial reduction (Fig. 3*A*). This shift suggests the formation of rGO from GO, mediated by biological reduction of *S. oneidensis* MR-1. The microbially reduced GO was dropped onto the carbon paper to form a thin layer, resulting in an rGO-coated cathode for subsequent microbial H_2_ electrosynthesis. An increase in biomass content on the rGO-coated electrode was detected compared to the control carbon paper (Fig. 3*B*), which was attributed to the large surface area of graphene. We further compared their electron uptake rates, demonstrating that the current consumption per milligram of cell protein was enhanced from 0.00464 μmol electrons/μg/h to 0.00636 μmol electrons/μg/h (Fig. 3*C*). This indicates that the introduction of graphene oxide increased conductivity, accelerating the electron-transfer processes between the cells of *S. oneidensis* MR-1 and rGO sheets. The H_2_ production yield increased from 7.82 μmol/mg/d to 25.69 μmol/mg/d (Fig. 3*D*). Then, rGO-coated biocathode with *S. oneidensis* MR-1 was further modified by replacing it with FeS nanoparticles functionalized *S. oneidensis* MR-1. In the presence of FeS nanoparticles, the biomass content, electron uptake rate, and hydrogen yield were further enhanced to 270.33 μg protein/cm^2^, 0.00817 μmol electrons/μg/h, and 43.11 μmol/mg/d (Fig. 3), respectively, suggesting that nanomaterial engineering significantly enhanced the microbial H_2_ electrosynthesis.

### Increasing Proton Pumping into the Periplasm by Expression of GR and CAN.

**Fig. 4.**
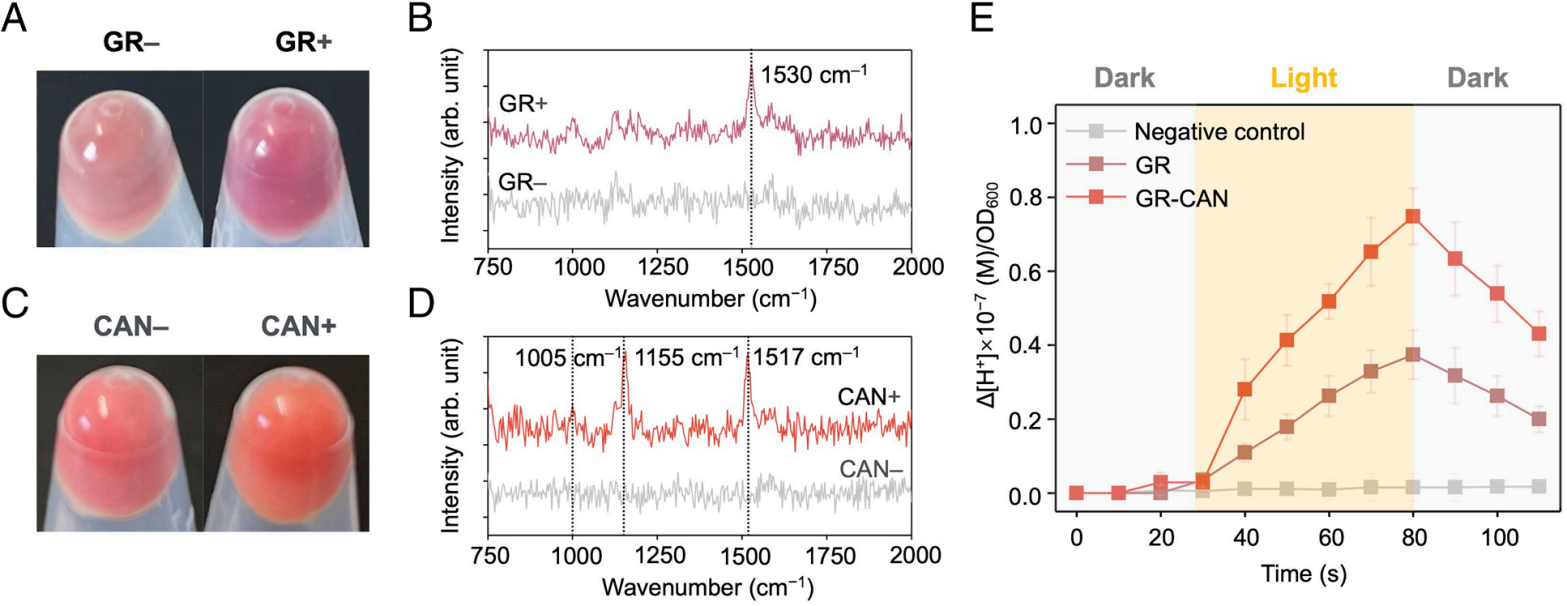
The expression of GR and CAN in *S. oneidensis* MR-1-GR-CAN enhanced H_2_ production boosted by the light. (*A*) The pictures of cell pellets with (GR+) and without (GR–) *Gloeobacter* rhodopsin expression. (*B*) A typical Raman spectrum of a cell with GR complex identified by a band at ~1,530 cm^–1^. (*C*) The pictures of cells pellet with (CAN+) and without (CAN–) canthaxanthin expression. (*D*) A typical Raman spectrum of a cell with CAN identified by a band at ~1,005, 1,155, and 1,517 cm^–1^. (*E*) Extracellular proton concentration changes in response to illumination with white light (~200 μmol/m^2^/s). Data are means ± SD, n = 3.

Outward proton-pumping rhodopsin is the most abundant microbial rhodopsin in nature, which has been widely used as a synthetic biology tool (35, 36). Bacteria with rhodopsin can harvest light energy to pump out protons into the periplasm, generating a proton gradient. Herein, we choose GR, from *Gloeobacter violaceus* because of its high turnover rate, broad pH range, and fast photocycling rate (37). The plasmid pLO11a-GR was transferred into *S. oneidensis* MR-1, resulting in the strain *S. oneidensis* MR-1-GR. The expression of GR was induced by 1 mM arabinose, leading to the cell pellet of the induced group displaying a pink color, in contrast to the uninduced control (Fig. 4*A*). Single-cell Raman spectroscopy identified a distinctive GRl band at around 1,530 cm^–1^ in arabinose-induced *S. oneidensis* MR-1-GR but not in the uninduced control. This is consistent with the previous reports (28, 38) (Fig. 4*B*). At the single-cell level, around 95% of the cell population were counted containing GR (number of measured cells n = 198, *SI Appendix*, Fig. S4*A*), indicating the gene expression of GR in most *S. oneidensis* MR-1-GR. A plate reader was employed to scan the absorbance of the cell extracts which detected the obvious absorbance at ~540 nm compared to the control group, further confirming a strong light absorption of GR in *S. oneidensis* MR-1-GR (*SI Appendix*, Fig. S5). Previous studies demonstrated that GR could be combined with carotenoids such as CAN and echinenone to enhance the proton-pumping capacity (35, 39). In this study, *S. oneidensis* was engineered to synthesize CAN. The expression of CAN turned the color of the cell pellet of *S. oneidensis* MR-1-GR-CAN (*SI Appendix*, Table S1), shifting it from initial red color, characteristic of WT *S. oneidensis* (40), to an orange-red color (Fig. 4*C*). Single-cell Raman spectroscopy confirmed the characteristic bands for CAN at around 1,005, 1,155, and 1,517 cm^–1^ (Fig. 4*D*) which are consistent with the Raman spectra of the commercially pure CAN reported by the previous study (35). Single-cell Raman analysis confirms that ~99% population of *S. oneidensis* MR-1-GR-CAN expressed CAN (number of measured cells n = 198, *SI Appendix*, Fig. S4*B*). A previous study has demonstrated that the proton pumping capacity of GR was significantly enhanced when assembled with CAN, exhibiting a 1.5 to 5 times increase compared to GR alone (41). To investigate the proton pumping capacities of cells with GR and GR-CAN, the pH dynamic change of the cell suspension in an unbuffered solution was examined. Light was turned on for 50 s and then off when pH remained stable. The proton pumping rate of *S. oneidensis* MR-1-GR-CAN, based on the change in extracellular proton concentration per OD_600_, was twice that of *S. oneidensis* MR-1-GR (Fig. 4*E*). This result suggests that the introduction of CAN antenna could effectively enhance the proton pumping efficiency of the rhodopsin-expressing cells.

### Enhanced H_2_ Production through Efficient Interactions of Reactants within Periplasmic Nanoreactor.

**Fig. 5.**
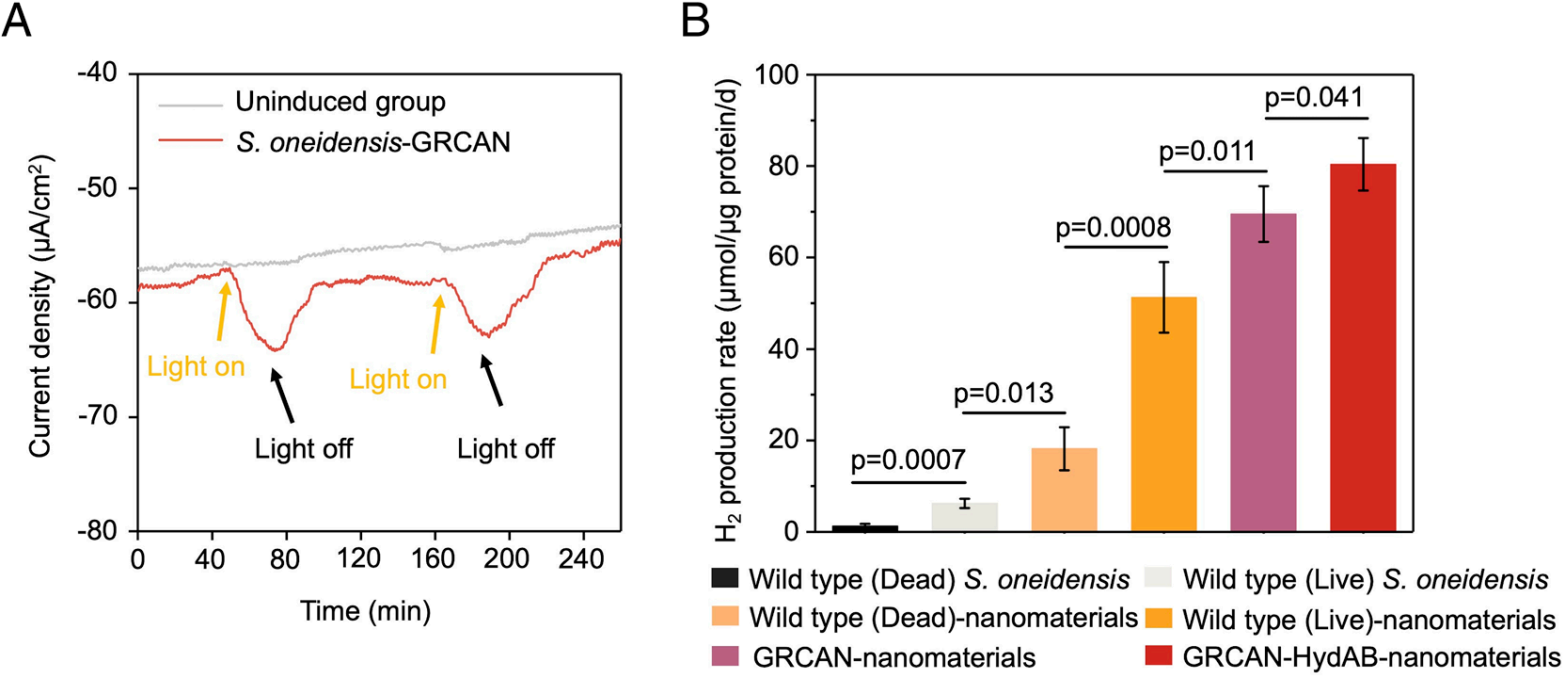
The integration of nanomaterials (rGO and FeS) and biological GR, CAN and [FeFe]-hydrogenase into the periplasmic bionanoreactor of *S. oneidensis* MR-1-GR-CAN-HydAB has achieved high H_2_ production. (*A*) The response of the engineered *S. oneidensis* MR-1-GRCAN to light and dark. (*B*) H_2_ production by dead *S. oneidensis* WT cells (after 15-min 110 °C heat inactivation), WT cells, WT cells (dead) with nanomaterial engineering, WT cells (live) with nanomaterial engineering, the engineered *S. oneidensis* MR-1-GR-CAN with GR-CAN, and the engineered *S. oneidensis* MR-1-GR-CAN-HydAB with GR-CAN and the overexpression of hydrogenase, at the potential of −0.75 V vs. SHE under illumination. Statistics were performed with Student’s *t*-test (data are means ± SD, n = 4).

The working electrode was set at a cathodic potential of −0.75 V vs. SHE to investigate the response of the cathode coated with *S. oneidensis* MR-1-GR-CAN to light. The cathode chamber was equipped with white LED lights to activate proton pumping in the GR-CAN complex of *S. oneidensis* MR-1-GR-CAN. The response of the cathodic current to the light was tested by switching on and off the LED lights. In the light, the current consumption was significantly enhanced compared to those in the dark conditions. The current density was boosted from −58 μA/cm^2^ to −64 μA/cm^2^ within 40 min (from 40 to 80 min in Fig. 5*A*), indicating the proton motive force generated by GR enhanced the electron uptake by the engineered cells. To investigate the electron uptake capacity of the engineered cells with *S. oneidensis* MR-1-GR-CAN in the light, strains were pregrown and inoculated into bioelectrochemical systems with rGO and functionalized with self-assembling FeS nanoparticles (Fig. 1). Then, we compared the hydrogen production in the nanoelectrochemical system with and without expression of GR-CAN in *S. oneidensis* MR-1-GR-CAN. At a potential of −0.75 V vs. SHE, the hydrogen yield of the microbial electrochemical system was increased by 35.6% (Fig. 5*B*). This was attributed to two major advantages of the GR-CAN. One was that the proton gradient generated by the GR-CAN complex promoted the electron transfer rate (Fig. 5*A*). Second, enhanced pumping of protons into the periplasmic nanoscale space created a favorable environment by elevating the concentration of protons and facilitating the hydrogenase-catalyzed interaction for H_2_ synthesis. Based on thermodynamic analysis of the hydrogen synthesis (*SI Appendix*, Fig. S6), the increased proton concentration led to a decrease of the Gibbs free energy, lowering the energy barrier for the conversion of protons to hydrogen. Therefore, the periplasmic bionanoreactor significantly boosted the generation of H_2_.

Furthermore, the overexpression of enzymes concentrated in the periplasmic environment also accelerated the reaction. Hydrogenases of *S. oneidensis* MR-1 were efficient enzymes for catalytic H_2_ turnover (42). Here, the [FeFe]-hydrogenase encoded by *hydA* (the large subunit, ~44 kDa) and *hydB* (the small subunit, ~12 kDa) were overexpressed, as confirmed by sodium dodecyl sulfate polyacrylamide gel electrophoresis (SDS-PAGE) analysis (*SI Appendix*, Fig. S7), to further improve hydrogen production. The result demonstrates that the hydrogen yield of hydrogenase-overexpressing strain *S. oneidensis* MR-1-GR-CAN-HydAB achieved 80.4 ± 5.8 μmol/mg protein/d, which is more than 10 times that of 7.8 ± 1.8 μmol/mg protein/d achieved by the WT *S. oneidensis* without modification, and more than four times than that of 18.2 ± 4.7 μmol/mg protein/d achieved by the abiotic control with FeS and rGO (Fig. 5*B* and Table 1). The hydrogen production by the rGO biocathode coated with *S. oneidensis* MR-1-GR-CAN-HydAB functionalized with FeS, compared to the control cathode, is shown in a movie in Movie S1. Compared to other similar approaches, this hydrogen production rate surpasses the traditional systems based on bacterial catalysts, as shown in Table 1. The bionanoreactor significantly outperformed the electrode with a low platinum load (0.03 mg/cm^2^ Pt) (FuelCellStore, UK) (*SI Appendix*, Fig. S8). Although there remains a performance gap between the bionanoreactor (0.27 mg/cm^2^ of protein) and the traditional platinum electrode (FuelCellStore, UK) loaded with 0.2 mg/cm^2^ of platinum catalyst (*SI Appendix*, Fig. S8), the biohybrid system offers a cost-effective and sustainable alternative. Its future potential hinges on further enhancements in biocatalytic cell loading density and the efficiencies of electron and proton transport. The Faraday efficiency of this bionanoreactor system engineered in *S. oneidensis* MR-1-GR-CAN-HydAB achieved 80.5% ± 6.8% within two days, a significant improvement from 14.0% ± 2.9% efficiency observed in the WT strain (*SI Appendix*, Fig. S9). Furthermore, when a potential of −0.85 V was applied, the hydrogen production rate of the bionanoreactor increased to 123.55 ± 25.9 μmol/mg protein/d (Table 1), despite a minor amount of hydrogen also detected in the abiotic materials (*SI Appendix*, Fig. S10). Notably, the majority of the engineered *S. oneidensis* cells maintained viability under both electrochemical conditions (−0.75 V and −0.85 V) (*SI Appendix*, Fig. S11). Previous research has reported the important role of rhodopsin in enhancing the survival of *S. oneidensis* MR-1 (36). Additionally, it has been demonstrated that the electrode-to-nanomaterial/electron mediator-to-hydrogenase pathway remains functional even in nonviable cells (43, 44), suggesting the robustness and versatility of the biohybrid system.

**Table 1.** Comparison to other similar microbial electrochemical systems for H_2_ production

Strains	Synthetic biology	Electrode materials	Abiotic materials	Cathode potential (vs. SHE)	H_2_ yield	Reference
*E. coli* BL21	Hydrogenase	Carbon paper (CP)	Methylviologen	~−0.9	~7.34 μmol/mg/d*	(44)
*S. oneidensis* MR-1	/	Carbon felt	/	−0.758	24.5 mL/m^3^/d	(14)
*S. oneidensis* MR-1	/	Carbon felt	/	−0.76	~4.6 μmol/mg/d	(16)
*S. oneidensis* MR-1	/	CP	/	−0.75	7.82 μmol/mg/d	This study
*S. oneidensis* MR-1	GR-CAN, hydrogenase	CP with rGO	FeS nanoparticles	−0.75	80.37 μmol/mg/d	This study
*S. oneidensis* MR-1	GR-CAN, hydrogenase	CP with rGO	FeS nanoparticles	−0.85	123.55 μmol/mg/d	This study

^*^The result was calculated according to 462.7 μmol/d and 0.3 g wet cells reported in ref. 44.

## Discussion

This study introduces and demonstrates the concept of a periplasmic bionanoreactor for sustainable and efficient hydrogen generation through water splitting. By integrating the abiotic nanomaterials and biological components, we have significantly improved biosynthetic reactions within a periplasmic bionanoreactor. In some species, such as *Rhodopseudomonas palustris* (45) and *Rhodobacter capsulatus* (46), their metabolic strategies lead to hydrogen synthesis occurring in the cytoplasmic space where cytoplasmic nitrogenases catalyze the reduction of protons with the electrons derived from ferredoxin (45, 46). In contrast, *S. oneidensis* relies on periplasmic hydrogenases for hydrogen synthesis metabolism (15). This approach offers two primary advantages. First, hydrogenases exhibit significantly higher turnover rates than nitrogenases; for example, [FeFe]-hydrogenases can achieve turnover rates up to 1,000 times higher than those of nitrogenases (47, 48). Second, the confined space of periplasm facilitates the effective interactions of substrates and enzymes at the nanoscale (24). Moreover, in the bionano H_2_ production system of this study, the periplasm provides an ideal environment for proton-pumping rhodopsin to significantly enhance hydrogen synthesis. According to the theory of thermodynamics, Gibbs free energy under general conditions (ΔG) is associated with the proton concentration ([H^+^]), expressed as:

[1] $$\Delta G=\Delta G^{0}+\text{RT}\times \text{ln}\frac{1}{{\left[H^{+}\right]}^{2}},$$

where ΔG^0^ is Gibbs free energy under the standard conditions, R is universal gas constant and T is temperature in Kelvin. The volume of the periplasmic space is estimated to be 8 to 16% of the entire cell (49, 50). Hence, the same amount of protons contributing to the concentration change in the periplasm is 5.2 to 11.5 times higher than in the cytoplasm. With rhodopsin pumping protons from cytoplasm to periplasm, the elevated proton concentration in the periplasm lowers Gibbs free energy and enhances interactions of hydrogenase with protons for efficient hydrogen synthesis.

In this periplasmic hydrogen synthetic system, both the outer and inner membranes play crucial roles, such as regulating proton movement with distinct permeabilities to protons. Although the outer membrane acts as a barrier to many molecules, including protons, cells can regulate proton transport across the cell envelope through passive diffusion via outer membrane-bound porins, thus maintaining relatively stable intracellular pH (51). The inner membrane selectively allows proton movement through active transport mechanisms mediated by various membrane proteins such as ion channels, transporters, and ATPases (52, 53). In this study, we propose that the increase in proton concentration in the periplasm, driven by rhodopsin pumping, leads to two significant outcomes (*SI Appendix*, Fig. S12). First, it promotes bacteria to regulate pH via hydrogen synthesis. The consumed periplasmic protons are replenished from the external environment where water splitting serves as a source of proton regeneration. Second, some protons may return to the cytoplasm via inner membrane protein (e.g., ATP synthase) to maintain pH homeostasis. The dynamic pH regulation across the membranes supports proton movement and supply within the periplasm, ensuring the maintenance of the proton gradient. This system could be extended for other reductive hydrogenation reactions in the periplasm such as the synthesis of formate (11) and succinate (54). The abiotic materials used in this work, including the microbially reduced GO and FeS nanoparticles, were synthesized by biological methods, making them more eco-friendly than traditional borohydride reduction approaches.

Biogenic FeS nanoparticles play an important role in enhancing electron-transfer processes (55). *S. oneidensis* MR-1 can naturally synthesize and assemble FeS nanoparticles, similar to the natural processes observed in sulfate-reducing bacteria (SRB). In nature, 97% of sulfides are produced by SRB (56). Most SRB can synthesize FeS nanoparticles by using ferrous compounds and sulfide (57). SRB assembled with FeS nanoparticles on the membrane and periplasm become electroactive due to their electrical conductivity and interaction with biological components (55). The reductive capability of FeS nanoparticles also makes it a suitable mediator in the microbial electrosynthesis system. In fact, molecular ensembles of iron and sulfide are common in biological systems. For example, iron–sulfur (Fe–S) clusters are involved in important biochemical pathways, such as photosynthesis and respiration (58). Interestingly, the Fe–S clusters are important structures of hydrogenase which act as a connective line to deliver electrons to the central active site (42). Inspired by the natural occurrence of FeS clusters and interconnected chains of Fe–S in hydrogenases, we introduced the FeS nanoparticles to the bionanoelectrochemical system which showed a significant enhancement in electron transfer and H_2_ production by serving as electron transfer chain in periplasm.

The application of synthetic biology to sustainable engineering provides significant means for improving microbial synthesis performance. Microbial rhodopsins represent one of the major photosystems in nature, converting light energy to biochemical energy (59). Among them, outward proton-pumping microbial rhodopsin has become an increasingly popular target for introduction into nonnative bacteria to supply energy, by generating a proton gradient across the cell membrane in the light (27, 35, 60). The proton motive force generated by the proton-pumping microbial rhodopsin has been demonstrated to increase intracellular ATP levels (61), survival rate (38), biomass growth (28), and bioproduction yield (27). In this study, by creating an electron sink via H_2_ generation, the light-activated GR combined with CAN not only enhanced the electron uptake rate of cells but also provided favorable conditions in periplasm with elevated proton content. While the introduction of rhodopsin boosted the H_2_ synthesis, the underlying mechanism remains elusive. For example, the dynamics of proton flux across the membranes are unclear. Practical application of the microbial photoelectrochemical system may require even more efficient rhodopsins. Therefore, we anticipate using artificial intelligence to support the design of rhodopsins with superior properties, such as high quantum efficiency (62), enhanced pH tolerance (63), and broad spectrum of light absorbance (64).

## Materials and Methods

### Bacterial Strains, Culture Conditions, and Plasmid Construction.

All bacterial strains and plasmids used in this study are shown in *SI Appendix*, Table S1. *E. coli* strains were grown in Lysogeny Broth (LB) at 37 °C under aeration by shaking at 200 rpm. *S. oneidensis* strains were grown in LB broth at 30 °C under aeration by shaking at 150 rpm. If required, 25 µg/mL chloramphenicol (Sigma-Aldrich) and 12.5 µg/mL tetracycline (Sigma-Aldrich) were added in the culture medium. All plasmid constructions were performed in *E. coli* DH5α. The plasmid pLO11a-GR contains the *Gloeobacter* rhodopsin gene. The plasmid pLO11a-GR-HydAB (*SI Appendix*, Fig. S13) contains the GR gene and the HydAB genes from *S. oneidensis* MR-1. The plasmid pAC-CANTHipi (Addgene: Plasmid #53301) contains the gene cluster for CAN synthesis (65). Induction of GR expression was accompanied by the addition of exogenous *trans*-retinal (Sigma-Aldrich) to achieve a final concentration of 5 μg/mL.

### Biosynthesis of FeS Nanoparticles.

*S. oneidensis* strains were precultured in LB at 150 rpm for 24 h. The cultured cells were collected by centrifugation at 3,000 g for 3 min and washed twice times with phosphate buffer saline (Sigma-Aldrich). Then, the cells were incubated in 100 mL anaerobic mineral medium with 0.5 mM Na_2_S_2_O_3_ and 20 mM sodium DL-lactate (Thermo Scientific), with an initial OD_600_ of 0.5, at 30 °C, stirring at 150 rpm. After 24 h incubation, 0.5 mM FeSO_4_ was added, and cultured for another 24 h to form FeS nanoparticles. The anaerobic mineral medium contained 5.85 g/L NaCl, 0.3 g/L NaOH, 1.5 g/L NH_4_Cl, 0.1 g/L KCl, 11.91 g/L [4-(2-hydroxyethyl)-1-piperazineethanesulfonic acid] HEPES, and 0.6 g/L NaH_2_PO_4_·H_2_O. The trace mineral minerals solution and amino acids solution were also added, and the compositions can be found in the previous study (13). Unless otherwise stated, all chemicals used above were purchased from Sigma-Aldrich. The FeS nanoparticles formation was operated under anaerobic conditions by pumping in pure nitrogen gas.

### Microbial Reduction of GO to Modify the Electrode.

For the reduction of graphene oxide, 5 mL of graphene oxide (3 mg/mL) solution was mixed with 10 mL of 100 mL of 20-h culture of *S. oneidensis* MR-1 strains. The mixture was shaken at 200 rpm, 25 °C. The microbially reduced GO was collected after 24 h. The rGO composite was washed three times by centrifugation and sonication with distilled water. Subsequently, it was dissolved in 10 mL of ethanol for further utilization. The CP (FuelCellStore) was cut into pieces with a surface area of 4 cm^2^. 2 mL of the microbially reduced GO solution was dropped on CPand air dried to form a GO-coated electrode. In the subsequent experiments, the biomass on the electrode was quantified by the bicinchoninic acid (BCA) assay kit (Thermo Scientific) (66). The procedure began by immersing the cathode CP in 5 mL of 0.2 M NaOH and vortexing for 1 min. Then, samples were heated at 95 °C in a heating block for 30 min to lyse cells. After cooling to room temperature, the samples were mixed with BCA reagents incubated at 37 °C for 30 min, and then, the absorbance at 562 nm was measured to determine the protein content.

### TEM Characterization.

*S. oneidensis* MR-1 cells with and without FeS nanoparticles were collected by centrifugation at 3,000 g for 3 min, then fixed in 4% formaldehyde (Sigma-Aldrich) and 2.5% glutaraldehyde (Thermo Scientific) in 0.1 M PIPES buffer (Sigma-Aldrich) at pH 7.2 for 1 h at room temperature ahead of storage at 4 °C. The following day samples were then washed with buffer (0.1 M PIPES buffer, pH 7.2), centrifuged, and the cell pellets enrobed in 2.5% low melting point agarose (Thermo Scientific). Dissected cubes (~1 mm^3^) of enrobed cells were treated with 50 mM glycine (Sigma-Aldrich) in buffer, then washed again in buffer ahead of secondary fixation with 1% (w/v) osmium tetroxide and 1.5% potassium ferrocyanide (Sigma-Aldrich) in buffer. Samples were washed extensively with Milli-Q water and stained with 0.5% uranyl acetate solution (Electron Microscopy Sciences) then washed again with Milli-Q water. The samples were then dehydrated through an ethanol series and infiltrated with and embedded in TAAB low-viscosity epoxy resin. The samples were then polymerized at 60 °C for 24 h. Sections of 90 nm were cut from the resin blocks using a Leica UC7 Ultramicrotome and collected onto 3 mm copper grids. The sections were then poststained with lead citrate and imaged using a JEOL Flash 120 kV TEM equipped with a Gatan Rio camera.

### SDS-PAGE to Validate Overexpression of the Periplasmic [FeFe]-Hydrogenase.

*S. oneidensis*-GR-HydAB strains were grown anaerobically in the anaerobic minimal medium as described above for 48 h at 30 °C, using 20 mM sodium DL-lactate (Thermo Scientific) as the electron donor and 10 mM sodium fumarate (Sigma-Aldrich) as the electron acceptor. After the anaerobic culture, cells were harvested and centrifuged (3,000 g, 4 °C, 15 min) to an OD_600_ of 5 in 10 mL. Cells were then washed in phosphate buffer saline (Sigma-Aldrich), centrifuged at 3,000 g, 4 °C for 15 min, resuspended in a buffer (100 mM Tris-HCl pH 8, 500 mM sucrose, 0.5 mM EDTA), incubated for 5 min on ice, and centrifuged at 3,000 g, 4 °C for 15 min. For recovery of periplasmic proteins, the pellets were resuspended in 1 mM MgCl_2_ and incubated for 2 min. The recovered periplasmic proteins were suspended in lithium dodecyl sulfate (Invitrogen). The samples were run in an 8 to 16% Tris-Glycine Gel (Thermo Scientific) at room temperature. All samples were standardized to contain 30 μg of protein, as quantified by BCA assay, and loaded in equal volumes of 42 μL. Electrophoresis was operated at 180 V for 50 min. Protein bands were stained using the Coomassie Blue staining protocol (67).

### Colony-Forming Units (CFU) Counting for Quantification of Viable Cells on the Electrode.

The electrode with biofilm was transferred to a 50 mL Falcon tube filled with 5 mL of sterile 0.9% NaCl solution and vortexed for 2 min to disperse the cells. A series of diluted solutions were spread onto LB agar plates, which were then incubated at 30 °C for 36 h before the CFU counting.

### Raman Spectroscopy Characterization.

Single *S. oneidensis* cells and FeS nanoparticles were characterized by Raman microspectroscopy. Before the measurements, samples were washed three times with distilled water to remove traces of culture medium and extracellular metabolites. 1.5-μL suspension was dropped onto an aluminum-coated slide and air dried for subsequent measurements. The cells were observed under a microscope after washing. Raman spectroscopic acquisition was performed using a LabRAM HR Evolution confocal Raman microscope using a 100×/0.75 air objective (HORIBA, UK). Single-cell Raman spectra were obtained using a 532-nm neodymium-yttrium aluminum garnet laser with a 300 grooves mm^–1^ diffraction grating and were acquired in the range of 100 to 3,200 cm^–1^. The laser power was set at ~80 mW which was attenuated by neutral density filters before focusing onto the samples. Spectra were recorded with LABSPEC 6 software (HORIBA, UK). All raw spectra were preprocessed within LABSPEC 6 software by cosmic ray correction, polyline baseline fitting and subtraction, and vector normalization of the entire spectral region.

### Linear Sweep Voltammetry (LSV) Analysis.

LSV tests were conducted on the CP-based electrode and biocathode with *S. oneidensis* cells. The polarization curves were obtained within a potential range between −0.85 V and −0.25 V vs. SHE with a scanning rate of 1 mV/s. The LSV was controlled by the potentiostat (PalmSens 4-channel Multi EmStat^3+^, Netherlands). The current was recorded, and the current density was calculated based on the current per square centimeter of the cathode projected area.

### Electrochemical Test for Fumarate Reduction.

The H-shaped dual-chamber reactor was constructed by connecting two chambers with a proton exchange membrane (PEM) Nafion membrane (*SI Appendix*, Fig. S14). The reference electrode (RE-5B, BASi) was Ag/AgCl in a 3 M KCl solution (+0.197 V vs. Standard Hydrogen Electrode, SHE). The working electrode was CP (4 cm^2^), and the counterelectrode was platinum wire. Precultured *S. oneidensis* suspensions were transferred to 200 mL minimal medium with 20 mM DL-lactate (Thermo Scientific) in 500 mL flasks. After 12-h growth at 30 °C with 150 rpm, the cell suspension concentration was adjusted to an OD_600_ of ~0.5 and injected into the working chamber with a working volume of 50 mL. In contrast, the counterchamber was filled with mineral medium without lactate and bacteria. The working chamber was purged with pure N_2_ gas to remove oxygen. The biocathode was imposed at −0.5 V (vs. Ag/AgCl) by the potentiostat (PalmSens, Netherlands). Once the cathodic current stabilized, 25 mM of fumarate was introduced to monitor the current change by the potentiostat (PalmSens 4-channel Multi EmStat^3+^, Netherlands).

### Proton Pumping Capacity Test of Engineered Cells in Light.

The precultured *S. oneidensis* strains were harvested and washed with an unbuffered solution containing salts (Sigma-Aldrich) as follows: 10 mM NaCl, 10 mM MgSO_4_·7H_2_O, and 0.1 mM CaCl_2_. Cells were then resuspended in the unbuffered solution to obtain an OD_600_ of ~1.5. The cell suspension was placed in the dark until the pH was stabilized, which was measured by a pH meter (Hanna edge). Following stabilization, the suspension was exposed to illumination with an intensity of ~200 μmol/m^2^/s for 50 s. Real-time pH values were recorded, and the corresponding proton concentration differential (Δ[H^+^]) was calculated.

### Light Response Test of Electrochemical Systems with Engineered Cells.

A photoelectrochemical system was constructed by surrounding the working chamber of the electrochemical system with 5 m of white LED light strips. The white LED light strips encircled the exterior of the working chamber to ensure even light distribution during the experiment (*SI Appendix*, Fig. S14). The strains with and without induction of GR-CAN complex were precultured as above and formed a biofilm on the electrode. The light was switched on for 40 min and then switched off. The currents were measured by the potentiostat (PalmSens 4-channel Multi EmStat^3+^, Netherlands).

### Cathodic Hydrogen Production.

The photoelectrochemical system was constructed as described above. The *S. oneidensis* cells were incubated in the working chamber for 48 h to form a biofilm on the electrode, with the addition of ~5 μM of riboflavin to facilitate the biofilm formation. Then, planktonic cells and mineral medium in the working chamber were removed and replaced with the medium containing 50 mM HEPES (Sigma-Aldrich) and 50 mM NaCl (Sigma-Aldrich). Then, the working electrode was set at −0.75 vs. SHE for 72 h. Hydrogen was detected in the headspace using gas chromatography (Shimadzu) equipped with the Carboxen-1010 column. For the light-driven hydrogen synthesis, LED light strips were switched on, providing illumination with a light intensity of approximately 200 μmol/m^2^/s. During the hydrogen production, the working chamber was maintained at 25 °C without agitation. For the abiotic control, the biocathode was heat-treated at 110 °C in an oven for 15 min to inactive the *S. oneidensis* MR-1 cells. In addition, the commercial electrodes made of carbon material coated with 0.03 mg/cm^2^ of platinum (FuelCellStore, UK) and 0.2 mg/cm^2^ of platinum (FuelCellStore, UK), respectively, were used as the cathode for the control experiments. Unless otherwise stated, all hydrogen production experiments were operated using the same electrolyte at 25 °C.

### Calculations.

Electron uptake rate (μ) of the cells could be calculated by the equation,

[2] $$\mu =\frac{\text{Electrons}\;\text{transferred}\;(\mu \text{mol})}{\text{Biomass}\;(\text{μg}\;\text{protein})\times \text{Time}\;(\text{hour})}.$$

Faraday efficiency (η) of the hydrogen formation could be calculated by the equation,

[3] $$\eta =\frac{\text{nzF}}{\text{Charge}\;\text{passed}\;(C)},$$

where *n* is the amount of hydrogen (mol), *z* is the number of transferred electrons (*z* = 2 for protons conversion to hydrogen), *F* is the Faraday constant (96,485 C/mol).

## Supplementary Material

Appendix 01 (PDF)

Movie S1. Movie to show hydrogen production catalysed by rGO and FeS functionalised *Shewanella oneidensis* MR-1-GR-CAN-HydAB attached to a carbon paper electrode and the control is a carbon paper electrode only.

## Data Availability

All study data are included in the article and/or supporting information.
