# Supplementary material for: Engineering bionanoreactor in bacteria for efficient hydrogen production
Source: Proc Natl Acad Sci U S A. 2024 Jul 10;121(29):e2404958121. doi: 10.1073/pnas.2404958121 (PMC11260135; doi:10.1073/pnas.2404958121)
Supplement: Supplementary file 1 — Appendix 01 (PDF) [file pnas.2404958121.sapp.pdf]

Supplementary information

Engineering bio-nanoreactor in bacteria for efficient hydrogen production

Weiming Tu, Ian P. Thompson and Wei E. Huang*

Department of Engineering Science, University of Oxford, Oxford OX1 3PJ, United Kingdom.

*Corresponding author: Wei E. Huang.

Email: wei.huang@eng.ox.ac.uk. Tel: +44 1865 283786.

Table S1. Strains and plasmids used in this study

**Strains**	**Comments**	**Reference**
E. coli DH5α	Commercially obtained (NEW England, UK)	Lab collection
Shewanella oneidensis MR-1	Wild-type strain	Lab collection
Shewanella oneidensis MR-1-GR	*Shewanella oneidensis* MR-1-GR harbouring pLO11a-GR	This study
Shewanella oneidensis MR-1-GR-CAN	*Shewanella oneidensis* MR-1-GR harbouring pLO11a-GR and pAC-CANTHipi	This study
Shewanella oneidensis MR-1-GR-CAN-HydAB	*Shewanella oneidensis* MR-1-GR harbouring pLO11a-HydAB-GR and pAC-CANTHipi	This study
Plasmids	**Comments**	**Reference**
pLO11a (Tc^r, RK2 *ori*, Mob^+)	Expression vector with P_{BAD} promoter and downstream cloning sites (gift from Oliver Lenz, Technische Universität Berlin, Germany).	(1)
pLO11a-GR	pLO11a containing the gene for GR rhodopsin from *Gloeobacter violaceus* PCC7421	(1)
pAC-CANTHipi	pAC-BETAipi with the gene cluster of the canthaxanthin synthetic pathway	(2)
pLO11a-HydAB-GR	pLO11a containing *GR* gene rhodopsin from *Gloeobacter violaceus* PCC7421, *HydA* and *HydB* genes from *Shewanella oneidensis* MR-1	This study

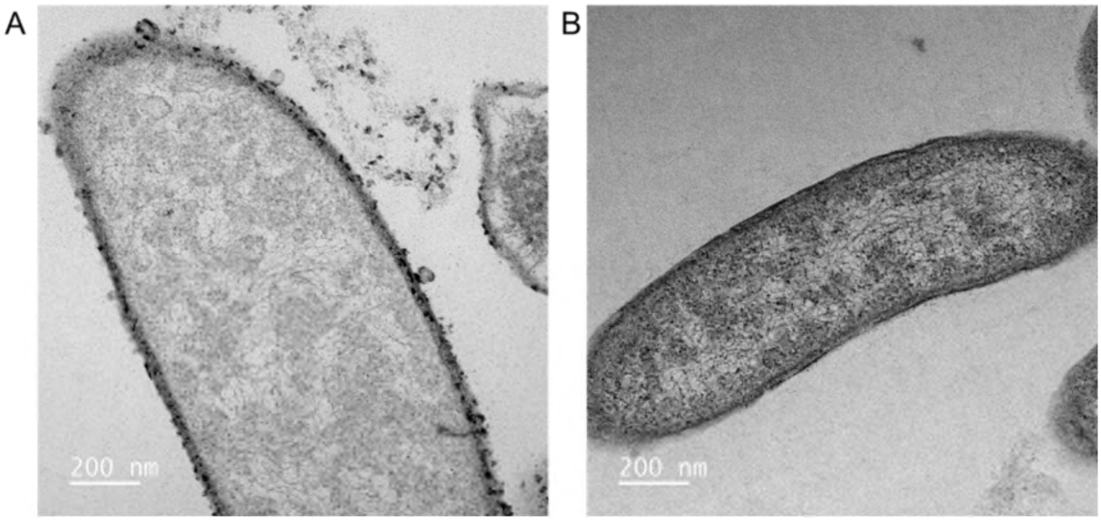

Fig. S1. TEM images of (A) *S. oneidensis* MR-1 with FeS nanoparticles, same as Fig.2A and (B) the control group without nanoparticles.

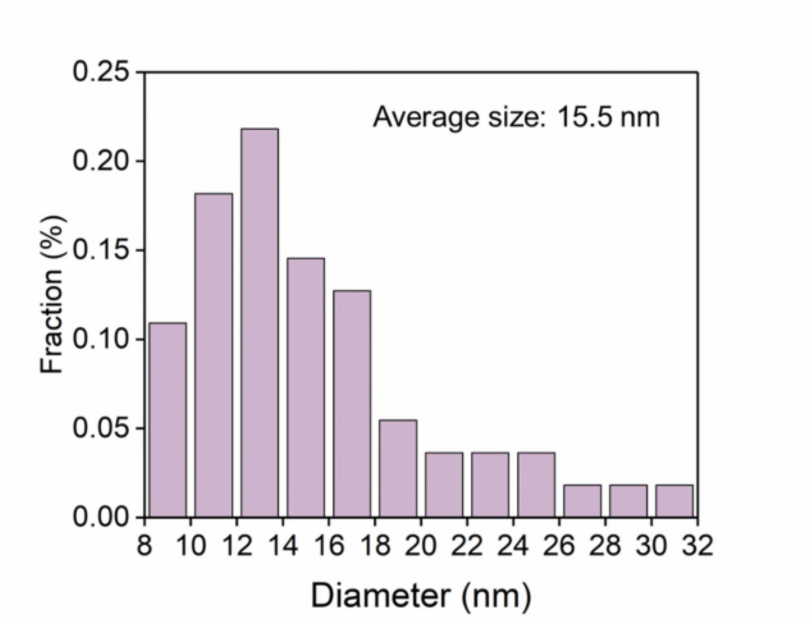

Fig. S2. Size distributions of FeS nanoparticles bio-synthesized by *S. oneidensis* MR-1 (n = 55).

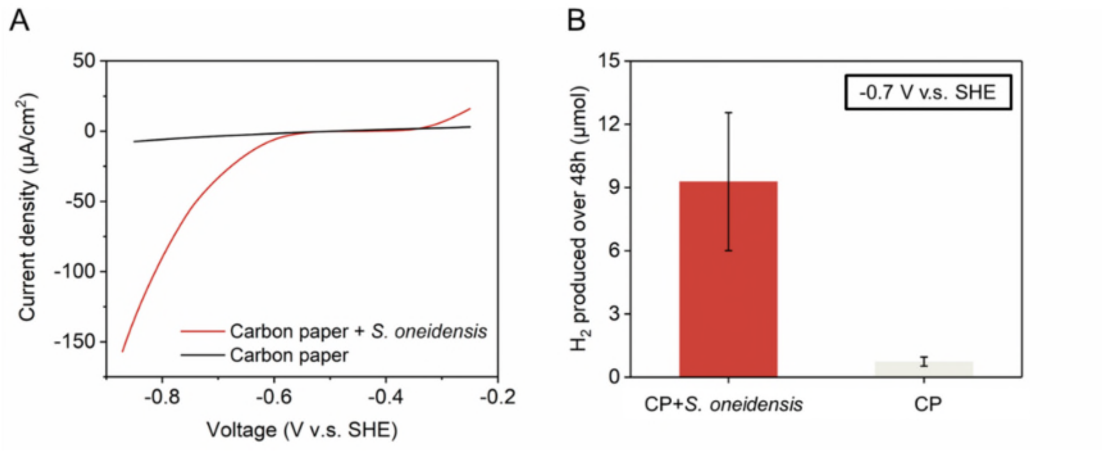

Fig. S3. Linear scanning voltammetry (LSV) analysis for the cathode of *S. oneidensis*. (B) Hydrogen production by carbon paper (CP) with and without *S. oneidensis* after 48 hours. Data are means ± SD, n = 3.

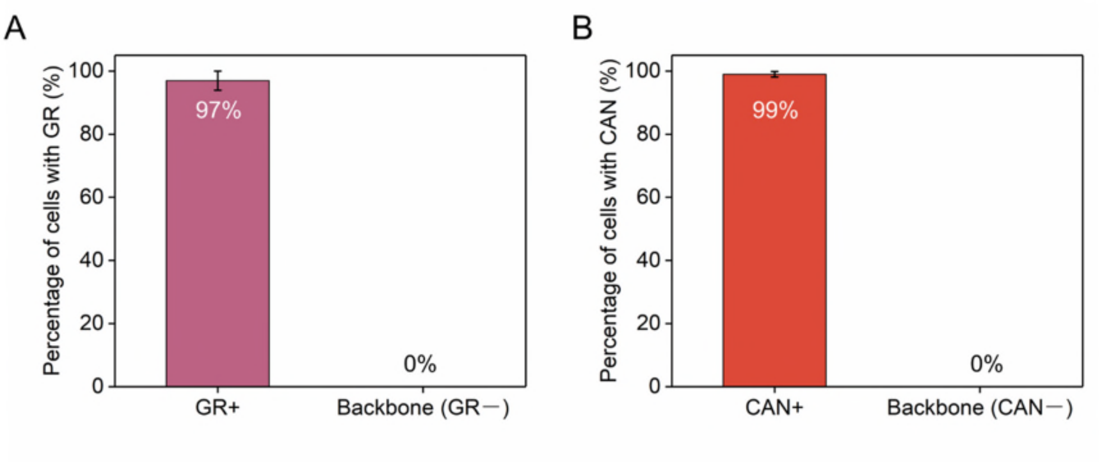

Fig. S4. Percentage of the cells expressing GR and canthaxanthin (CAN) in the population. Data are means ± SD, n = 3.

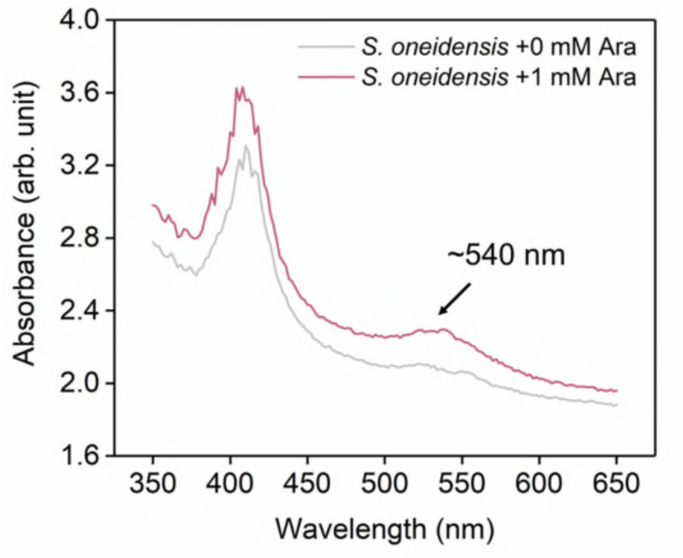

Fig. S5. Absorbance scan of the arabinose-induced *S. onidensis*-GR and noninduced cell extracts.

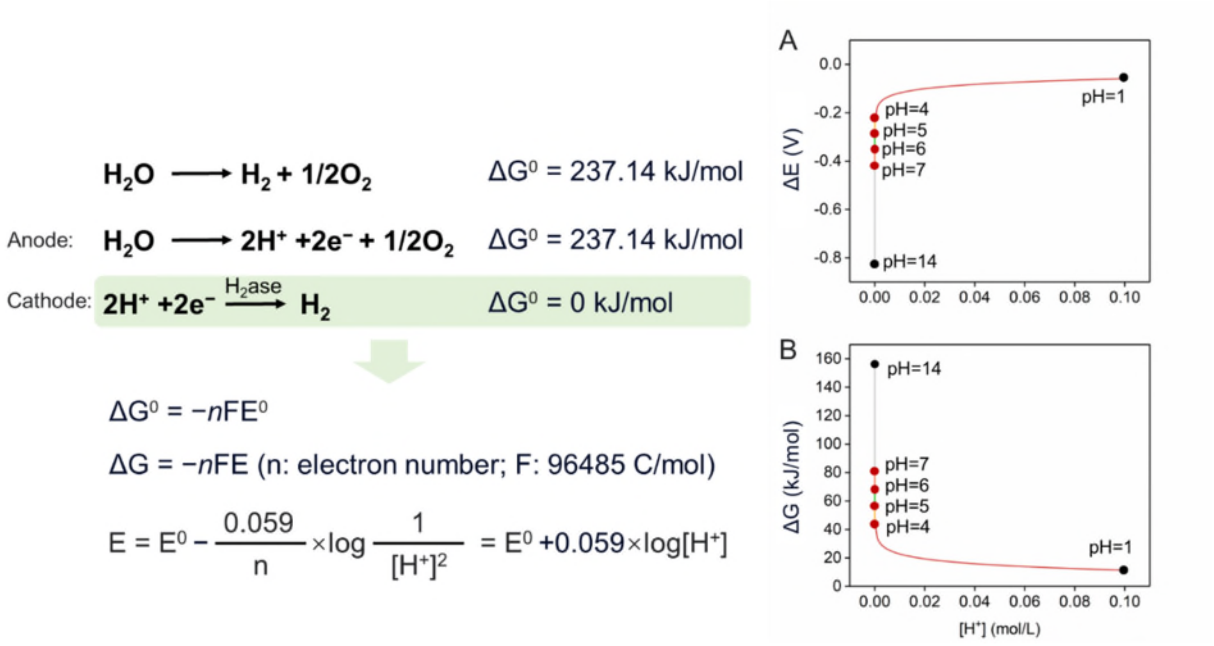

Fig. S6. Thermodynamic analysis of redox potential and Gibbs free energy of converting protons to hydrogen.

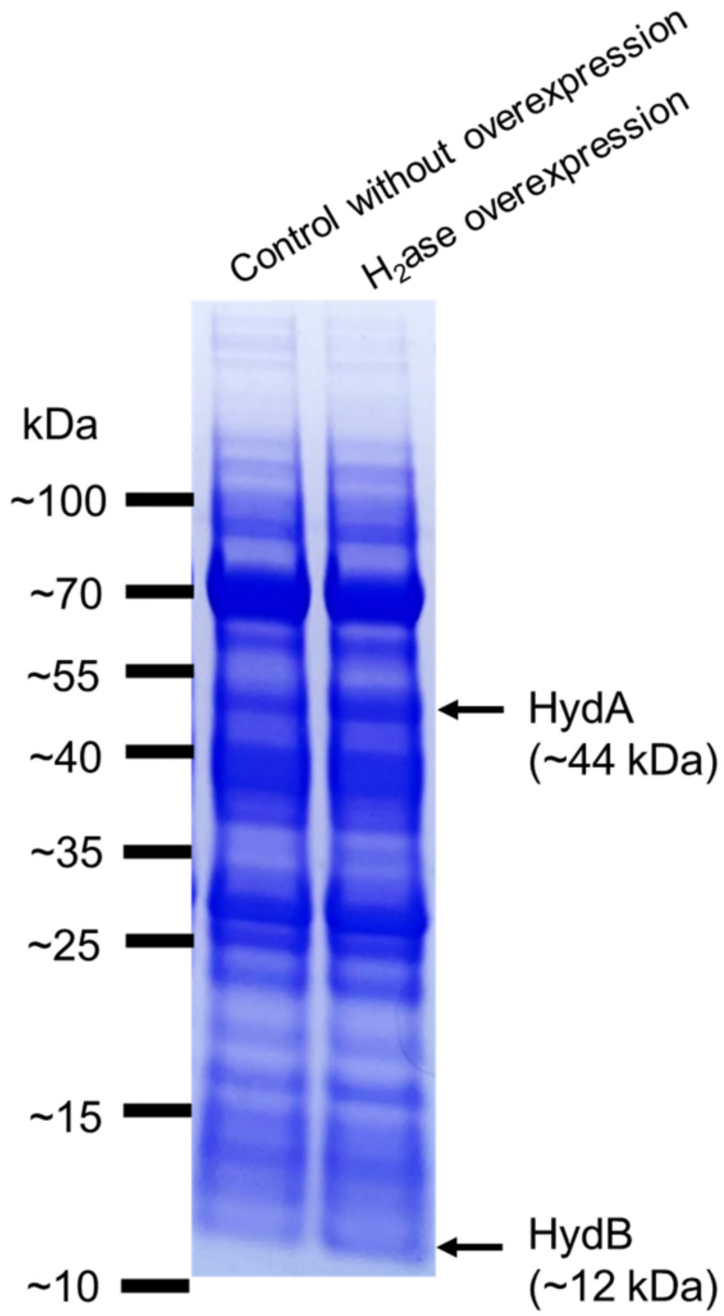

Fig. S7. Periplasmic proteins of the arabinose-induced (H_2ase overexpression) and uninduced *S. oneidensis* MR-1-GR-CAN-HydAB. The molecular masses of HydA and HydB peptides are predicted from the amino acid sequence.

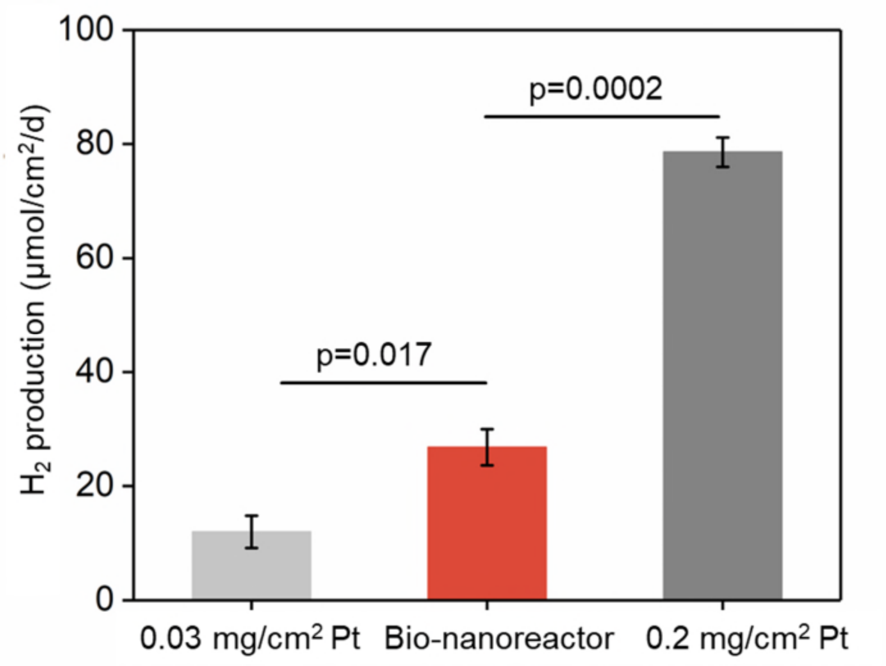

Fig. S8. Comparison of hydrogen production between the bio-nanoreactor and the electrodes with varying platinum loadings (0.03 mg/cm^2 Pt and 0.2 mg/cm^2 Pt) at 0.75V vs. SHE, pH 7.

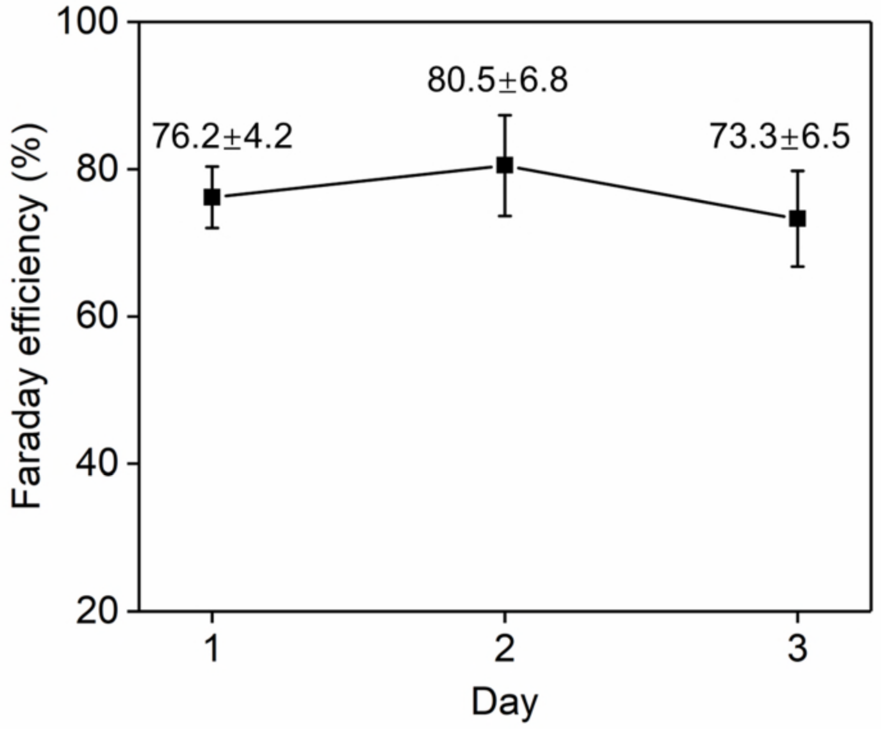

Fig. S9. Faraday efficiency of a three-day running of the photoelectrochemical bio-nanoreactor. Data are means ± SD, n = 3.

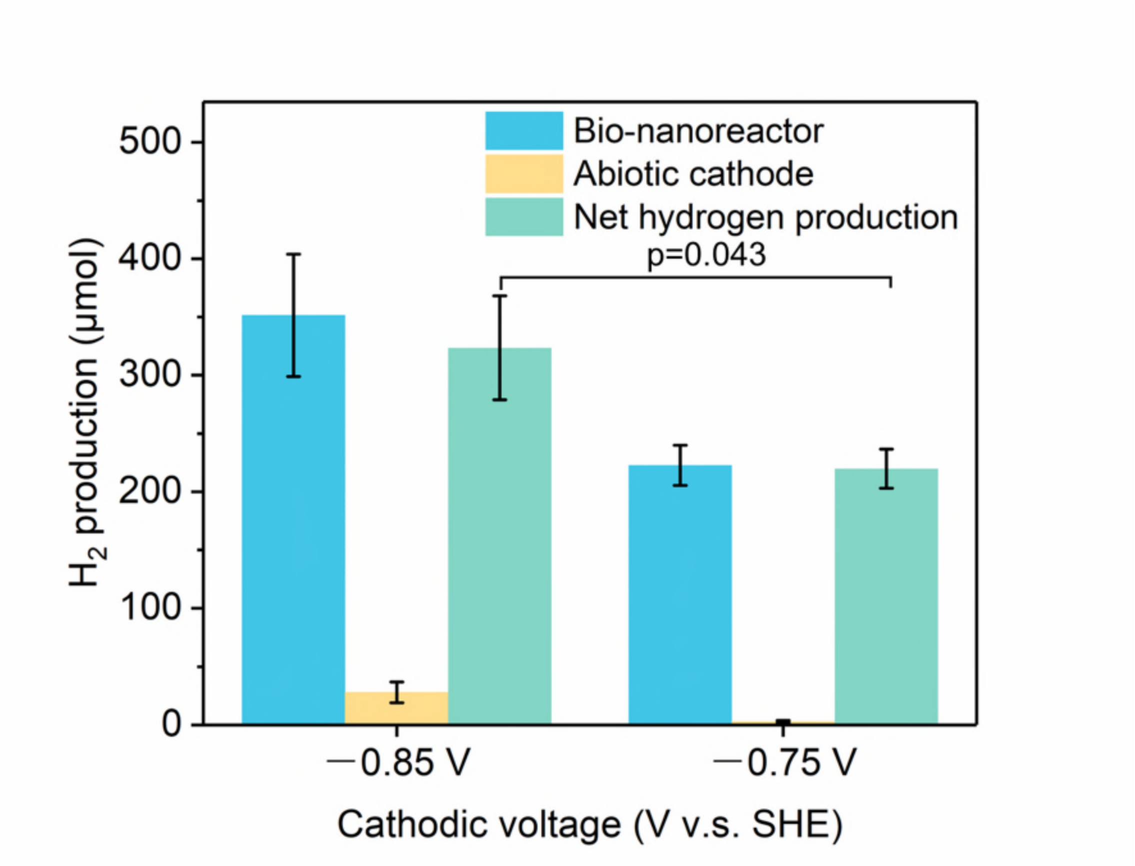

Fig. S10. Hydrogen production at the potential of −0.75 V and −0.85 V after three-day operations. Statistics were performed with Student's t-test. Data are means ± SD, n = 3.

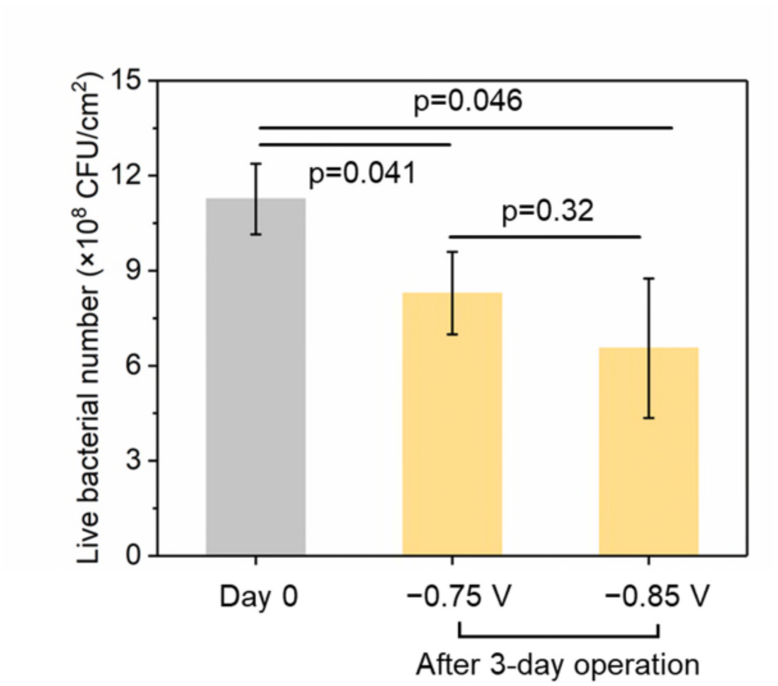

Fig. S11. Comparison of viable cells of the engineered *S. oneidensis* before and after three-day operation at different cathodic potentials. Statistics were performed with Student's t-test (Data are means ± SD, n = 3).

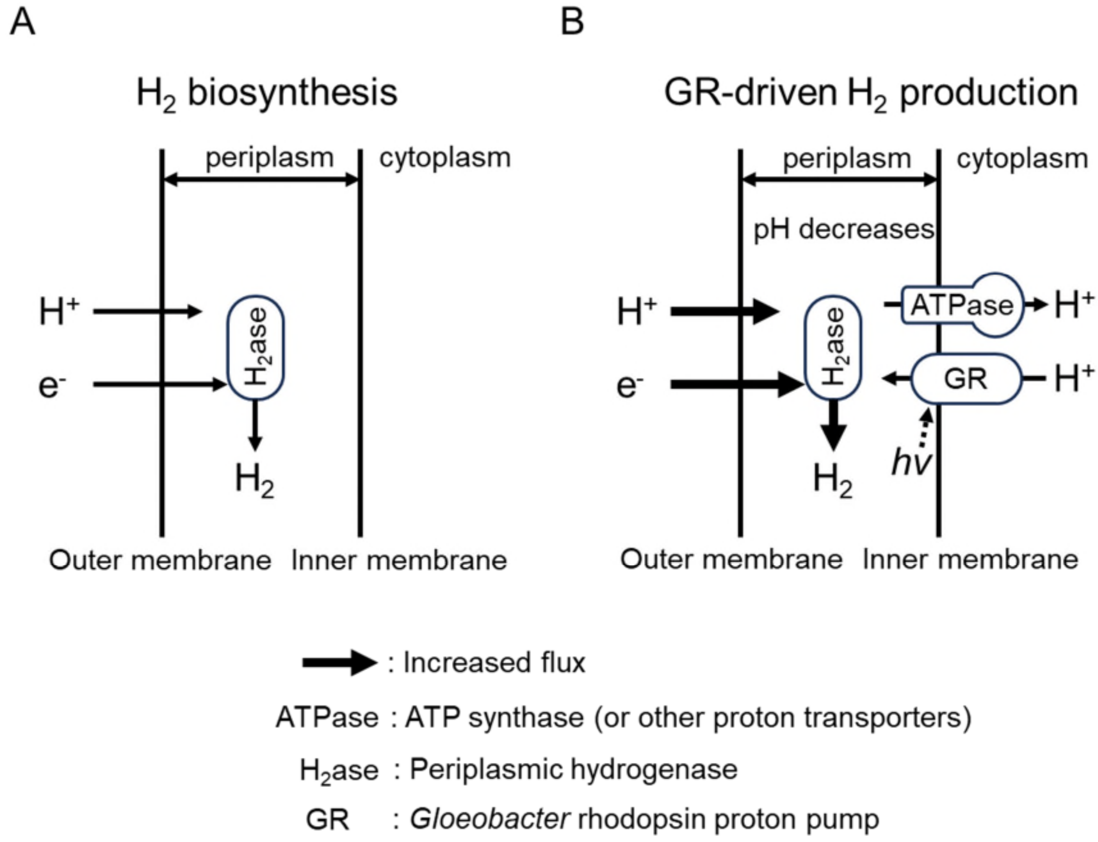

Fig.S12. Proposed periplasmic proton regulation for enhanced H_2 synthesis induced by GR.

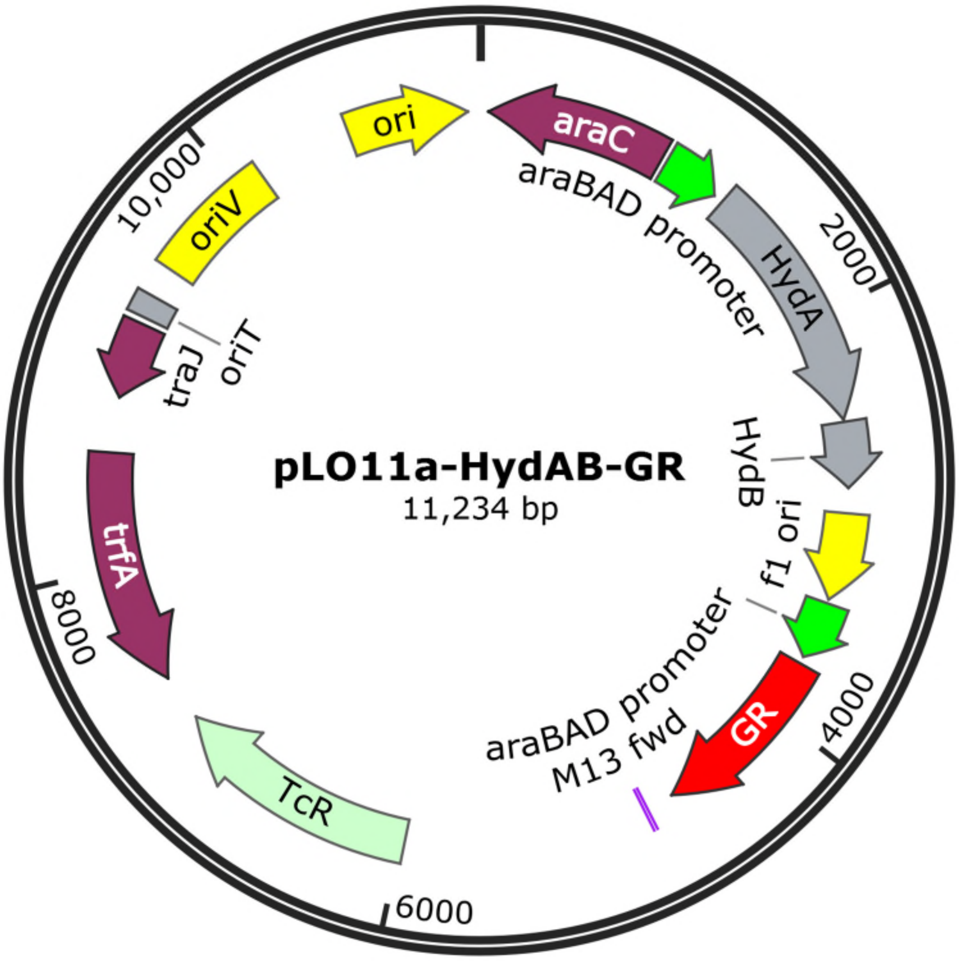

Fig. S13. Plasmid map of the pLO11a-HydAB-GR.

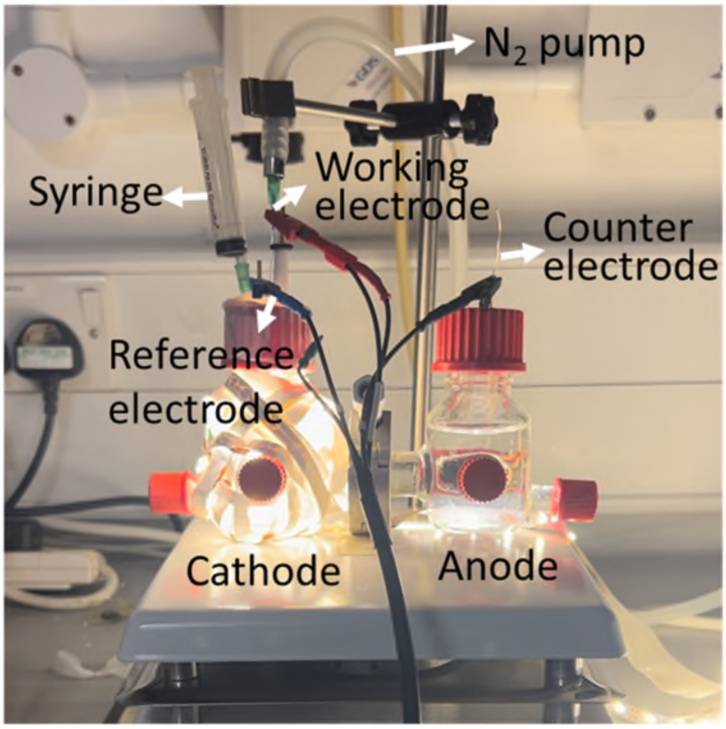

Fig. S14. Picture of the practical set-up of the water-splitting electrochemical system using *S. oneidensis*-based cathode.

Movie S1. Movie to show hydrogen production catalysed by rGO and FeS functionalised *Shewanella oneidensis* MR-1-GR-CAN-HydAB attached to a carbon paper electrode and the control is a carbon paper electrode only.

Reference

(1) Davison, P. A., Tu, W., Xu, J., Della Valle, S., Thompson, I. P., Hunter, C. N., & Huang, W. E. (2022). Engineering a rhodopsin-based photo-electrosynthetic system in bacteria for CO_2 fixation. ACS Synthetic Biology, 11(11), 3805-3816.

(2) Cunningham, F. X., & Gantt, E. (2007). A portfolio of plasmids for identification and analysis of carotenoid pathway enzymes: Adonis aestivalis as a case study. Photosynthesis Research, 92, 245-259.
